# Fluorescent Aptasensor for Highly Specific Detection of ATP Using a Newly Screened Aptamer

**DOI:** 10.3390/s22072425

**Published:** 2022-03-22

**Authors:** Xin Chen, Yangkun Feng, Haohan Chen, Yuting Zhang, Xiaoli Wang, Nandi Zhou

**Affiliations:** 1 The Key Laboratory of Carbohydrate Chemistry and Biotechnology, Ministry of Education, School of Biotechnology, Jiangnan University, Wuxi 214122, China; 6190203001@stu.jiangnan.edu.cn (X.C.); 7190201030@stu.jiangnan.edu.cn (H.C.); yutingzhang@jiangnan.edu.cn (Y.Z.); xiaoliwang@jiangnan.edu.cn (X.W.); 2 Medical College, Nantong University, Nantong 226007, China; ykfeng@njmu.edu.cn

**Keywords:** ATP, aptamer, graphene oxide, strand displacement amplification, molecular beacon, fluorescent sensor

## Abstract

Owing to the significant roles of adenosine triphosphate (ATP) in diverse biological processes, ATP level is used to research and evaluate the physiological processes of organisms. Aptamer-based biosensors have been widely reported to achieve this purpose, which are superior in their flexible biosensing mechanism, with a high sensitivity and good biocompatibility; however, the aptamers currently used for ATP detection have a poor ability to discriminate ATP from adenosine diphosphate (ADP) and adenosine monophosphate (AMP). Herein, an ATP-specific aptamer was screened and applied to construct a fluorescent aptasensor for ATP by using graphene oxide (GO) and strand displacement amplification (SDA). The fluorescence intensity of the sensor is linearly related to the concentration of ATP within 0.1 μM to 25 μM under optimal experimental conditions, and the detection limit is 33.85 nM. The biosensor exhibits a satisfactory specificity for ATP. Moreover, the experimental results indicate that the biosensor can be applied to determine the ATP in human serum. In conclusion, the screened aptamer and the biosensor have promising applications in the determination of the real energy charge level and ATP content in a complex biological system.

## 1. Introduction

As a high-energy compound, adenosine triphosphate (ATP) can be found in all living cells. It can provide energy directly for the organisms and plays important roles in various cell life activities and signal transmission [1,2]. The level of ATP reflects the energy charge status of the cells and is closely related to cellular metabolic processes. At the same time, ATP plays significant roles in various physiological and pathological processes, and its level is also related to many diseases. For example, an abnormal level of ATP in the body is linked to Parkinson’s disease and cardiovascular disease [3]. As an indicator, the measurement of ATP plays an important role in biochemical research and clinical diagnosis [4]. Currently, the main methods for ATP detection include bioluminescence [5], high performance liquid chromatography [6] and capillary electrophoresis [7], etc.; however, high costs, the high requirements for equipment and professional individuals, and a complicated pretreatment of samples are the defects of the above methods [8].

Aptamers are oligonucleotide sequences and can be screened by the technology of a systematic evolution of ligands with exponential enrichment (SELEX) in vitro [9]. The aptamers and the specific target substances can interact with a high affinity and specificity [10,11]. Currently, different aptamers specific for metal ions [12], small molecules [13], drugs [14], proteins [15] and cells [16] have been reported. The advantages of being inexpensive and having a high stability allow for aptamers to be widely used as molecular recognition elements in the fields of biosensing, molecular imaging, clinical diagnosis and other fields [17,18]. In 1993, Szostak et al. reported the RNA aptamer for ATP for the first time [19] and the first DNA aptamer for ATP was screened in 1995 [20]. Since then, most of the works on ATP biosensors have been carried out based on this DNA aptamer, while the ATP aptamer has been one of the model aptamers widely used in biosensing. In order to achieve a better application, this classic aptamer has even been modified in different forms. Li et al. mutated and modified the DNA aptamer by cutting off specific nucleotides on the aptamer and increasing the complexity of the aptamer structure [21]. Biniuri et al. mutated the bases of the classic ATP aptamer, predicted the binding sites of the aptamer for ATP by a molecular dynamics simulation, and obtained a higher affinity aptamer with the help of microscale thermophoresis [22]. Despite the great success of this aptamer in the construction of ATP biosensors, it cannot effectively distinguish the phosphate groups at the 5′ position of adenosine, that is, such biosensors cannot discriminate ATP from other adenylate derivatives, such as adenosine diphosphate (ADP), adenosine monophosphate (AMP) and cyclic adenosine monophosphate (cAMP) [21,23]. Therefore, the ideal aptamers with high specificity for ATP are eagerly desired to achieve an accurate evaluation of the energy charge level in organisms.

At present, aptamers are used to construct various types of biosensors, such as fluorescence aptasensors, electrochemical aptasensors and colorimetric aptasensors [24,25,26]. Among them, the fluorescence aptasensors have a high sensitivity and the experimental results are more intuitive [27]. Meanwhile, various types of aptasensors for ATP have also been reported [28,29,30], but the limitation of the aptamer in its specificity leads to the defection of sensors in their specificity.

High sensitivity is generally a basic requirement for biosensors. Signal amplification technology is frequently applied to improve detection sensitivity in the design of biosensors. Nucleic acid-based signal amplification technologies are widely used in the construction of aptasensors because of the nucleic acid properties of the aptamers, including strand displacement amplification (SDA) [31,32], rolling circle amplification (RCA) [33,34], hybridization chain reaction (HCR) [35], exonuclease-assisted amplification [36], catalytic hairpin self-assembly amplification (CHA) [37], etc. Among them, SDA is easy to operate and does not require repeated heating cycles. It enables DNA to be exponentially amplified in a short period of time. Therefore, SDA is widely used in the construction of sensors related to nucleic acids and aptamers [38].

In this study, an aptamer specific for ATP was screened, which can effectively discriminate ATP from ADP and AMP. Then this aptamer was employed as the recognition element in a finely designed functional chimera sequence to develop a fluorescent biosensor to detect ATP by using polymerase and endonuclease-based strand displacement amplification technology. The fluorescent biosensor showed a high performance for the target and was employed to determine real samples.

## 2. Materials and Methods

### 2.1. Materials and Reagents

Nb.bpu10I endonuclease and Bsm DNA polymerase were obtained from Thermo Scientific (Waltham, MA, USA). Graphene oxide (GO) was obtained from Aladdin Reagent Co., Ltd. (Shanghai, China). Deoxyribonucleoside triphosphate (dNTP), ATP, ADP, AMP, guanosine triphosphate (GTP), uridine triphosphate (UTP) and cytidine triphosphate (CTP) were all purchased from Sangon Biotech Co., Ltd. (Shanghai, China). Human serum was purchased from XinFan Bio-technology Co., Ltd. (Beijing, China). In all experimental procedures, ultrapure water (18.2 MΩ cm) acquired from a Micropore filtration system was employed to prepare all solutions.

In this work, all the DNA sequences were synthesized and modified by Sangon Biotech Co., Ltd. (Shanghai, China). In the Supplementary Information, these sequences are presented in Table S1.

### 2.2. Characterization of the Aptamer

The magnetic beads-based SELEX was carried out to screen the aptamer sequences specific for ATP and the procedure of the SELEX is described in the Supplementary Information. The performance of the aptamer sequences was characterized. To determine the dissociate constant (K_d_) of the aptamer, the 5′FAM modified aptamer was diluted with the binding buffer (50 mM Tris-HCl containing 5 mM KCl, 100 mM NaCl and 1 mM MgCl_2_, pH 7.4) to 200 μL with an ultimate concentration of 50–500 nM. The above system was heated at 95 °C for 10 min, followed by being rapidly ice-bathed for 10 min. An amount of 1 μL of 100 mM ATP was mixed and shaken at 25 °C for 100 min, then 20 μL GO was introduced. Following incubation for 40 min at 25 °C, the system was centrifuged at 12,000 rpm for 12 min. Finally, all the supernatants were placed in a 96-well plate and an EnSpire^®^ Multimode Plate Reader (PerkinElmer, Inc., Waltham, MA, USA) was used to detect the fluorescence intensity with a 488 nm excitation wavelength and 525 nm emission wavelength. Based on the equation: $y=\mathrm{Bmax}\times \left[\mathrm{free}\;\mathrm{ssDNA}\right]/\left(K_{d}+[\mathrm{free}\;\mathrm{ssDNA}]\right)$, the K_d_ was evaluated through non-linear fitting using the OriginPro 2021(64bit)9.8.0.200 software. Y is the fluorescence intensity, Bmax is the maximum binding sites number, and [free ssDNA] is the free ssDNA concentration.

The 5′FAM modified aptamer was diluted with a binding buffer to 200 μL with a final concentration of 300 nM to assess the specificity of the aptamer for ATP. The solution was incubated at 95 °C for 10 min, followed by being promptly ice-bathed for 10 min. After that, 1 μL of 100 mM ATP, ADP or AMP were added, respectively, then mixed and reacted at 25 °C for 100 min. Followed by the addition of 20 μL GO, the above system was reacted for 40 min at 25 °C. Following centrifugation at 12,000 rpm for 12 min, the supernatants were added to a 96-well plate and the EnSpire^®^ Multimode Plate Reader was used to detect the fluorescence intensity.

### 2.3. The Fluorescent Detection of ATP

The fluorescent measurement of ATP can basically be divided into three steps: an ATP-mediated release of the functional chimera sequence from GO; polymerase and endonuclease-catalyzed SDA; and the fluorescent signal recovery of the molecular beacon.

For the first step, a 2 μL 100 μM chimera sequence was added to the binding buffer, incubated at 95 °C for 10 min, followed by being promptly ice-bathed for 10 min. Following the introduction of 5 μL GO, the above system was reacted for 40 min at 25 °C with shaking. Subsequently, the mixture containing various concentrations of ATP and the above system was continuously incubated at 25 °C for 100 min with shaking. Once the ATP bound specifically to the aptamer region of the chimera, the sequence was dissociated from the GO and released into the solution. The supernatants were recovered as the template for strand displacement amplification after the centrifugation for 15 min at 12,000 rpm.

For the next step, 5 μL of the above supernatants were introduced into the system containing a 3 μM primer, 5 U Bsm DNA polymerase, 8 U Nb.bpu10I endonuclease, 250 μM dNTP and 1× Buffer R. The SDA reaction system was overall 30 μL. To obtain a single-stranded reporter probe, the above system was incubated for 90 min at 37 °C to perform SDA. To ensure the enzymes inactivation, the system was maintained at 80 °C for 20 min after the reaction. Polyacrylamide gel electrophoresis (PAGE) was employed for the verification of the obtained reaction products.

For the final step, a 1 μL 100 μM molecular beacon was mixed with 25 μL of the above strand displacement amplification products, and the mixture was diluted with the binding buffer to 100 μL. The denaturation of the above mixture was carried out at 95 °C for 5 min, then it was incubated at 25 °C for 90 min. The fluorescence intensity was detected at a 488 nm excitation wavelength and 525 nm emission wavelength using the EnSpire^®^ Multimode Plate Reader.

### 2.4. Polyacrylamide Gel Electrophoresis

The verification of the whole experimental process was carried out by performing a 12% PAGE. The prepared polyacrylamide gel was placed in a 1× TBE buffer (89 mM Tris, 89 mM boric acid and 2 mM EDTA·2Na, pH 8.0). Electrophoresis was performed at the voltage of 120 V for 50 min. GelRed was applied to stain the gel following the electrophoresis. After 45 min of staining, the gel was imaged with a UV imaging system.

### 2.5. Detection of ATP in Serum Samples

ATP spiked in human serum samples was employed to verify the practicability of the constructed ATP fluorescent aptasensor. Firstly, the supernatant obtained through the centrifugation of the human serum at 12,000 rpm for 10 min was diluted 10 times with the binding buffer. Subsequently, ATP-spiked serum samples could be obtained by mixing various concentrations of ATP with the pretreated serum. The ATP in human serum samples was measured in the same way as the standard ATP.

## 3. Results and Discussion

### 3.1. Screening and Characterization of ATP-Specific Aptamer

To obtain high specific aptamers for ATP, the magnetic beads-based SELEX technology was employed to perform the screening. Particularly, based on our previous study, for the screening of aptamers that specifically bind to small molecule targets, the modified DNA library-immobilized magnetic beads-based SELEX shows superiority [39]. The experimental procedure of SELEX is described in the Supplementary Information. The enrichment and selection processes were monitored by fluorescent recovery of the sequences. The screening reached saturation after 10 rounds of selection. To improve the specificity of the screened aptamers, counter screening was repeatedly carried out in the later rounds of selection by using ADP and AMP. Therefore, sequences with an affinity for both ATP, ADP and AMP were effectively removed.

**Figure 1:**
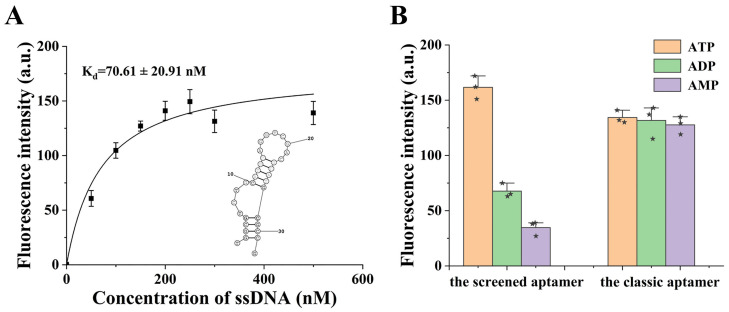
(**A**) The saturation curve and the dissociate constant (K_d_) of the aptamer. Inset shows the secondary structure of the obtained aptamer. (**B**) Specificity of the screened aptamer and the classic adenosine triphosphate (ATP) aptamer. The Asterisk represents the individual data points (n = 3).

The products after the final round of screening were analyzed by high-throughput sequencing and a candidate aptamer sequence was chosen from among the obtained sequences (data not shown). The sequence was further optimized by truncation, and a final sequence with the length of 32 nucleotides (see Table S1) was obtained and selected as the optimal aptamer for ATP. The performances of the aptamer were then characterized. According to the results of the saturation curve (Figure 1A), the dissociation constant (K_d_) was determined to be 70.61 ± 20.91 nM, which is remarkably lower than the classic ATP aptamer, indicating a higher affinity for ATP [20]. Moreover, the specificity of the aptamer could be evaluated through comparing the binding ability of the aptamer to ATP, ADP and AMP, respectively. As shown in Figure 1B, the aptamer shows significant difference in binding to different adenylates. The affinity of the aptamer for ATP was 2.4 and 4.7 folds higher than those of the ADP and AMP, respectively. Moreover, we tested the significance of the data using the SPSS 19.0 software—the results show that there was a significant difference between the affinities of the screened aptamer for ATP, ADP and AMP, and the difference was statistically significant (*p* < 0.001). The specific results of the descriptives and ANOVA of the data are shown in Table S2; however, due to the similar structures of these adenylate derivatives, it is difficult to obtain an ATP aptamer that was completely without an affinity for ADP and AMP. Although this aptamer still shows a low affinity for these interference compounds, the constructed biosensor can already be considered satisfactory in specificity. As a comparison, the specificity of the commonly used classic aptamer for ATP (Table S1) was also evaluated by using the same method. The results show that this aptamer can hardly discriminate ATP from ADP and AMP and the affinities for these three compounds are almost identical. Therefore, the screen aptamer was apparently predominant and was subsequently used for the construction of the fluorescent aptasensor for ATP.

### 3.2. Design of the Fluorescent Aptasensor for ATP

**Figure 2:**
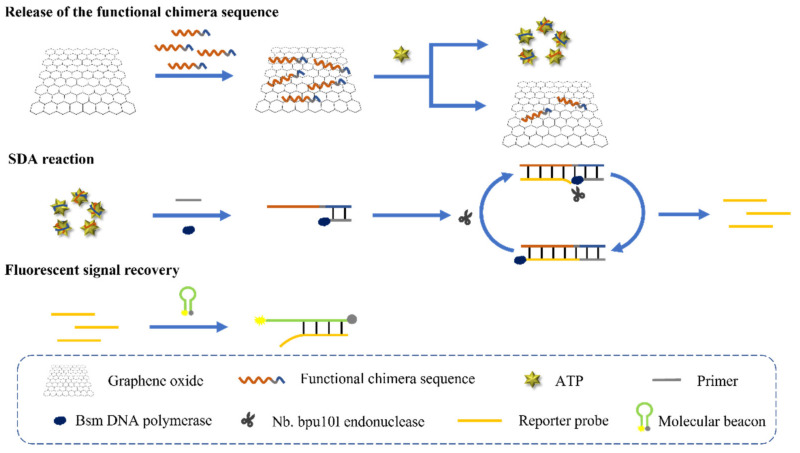
Schematic illustration of the fluorescence aptasensor for ATP.

Figure 2 demonstrates the mechanism of the fluorescent aptasensor for ATP. A functional chimera single-stranded DNA sequence was designed, which contained three regions. The first, an ATP aptamer region located at the 5′ end of the sequence served as the highly specific recognition element of the sensor (the red line segment in the functional chimera sequence). Second, an antisense sequence of the Nb.bpu10I cleavage site located in the middle of the sequence and finally the primer binding region located at the 3′ end of the sequence that facilitated the smooth progress of subsequent SDA and the production of a huge number of the reporter probes.

By π-π stacking, the single-stranded chimera sequence was adsorbed on GO. In the presence of ATP, the whole sequence conformation changed due to the binding between the ATP and the aptamer region of the sequence, which in turn led to the dissociation of the sequence from the GO. This step achieved the conversion of the detection of ATP into the DNA quantitative determination. Nevertheless, the sensitivity of the detection was limited in the absence of the effective signal amplification, because a certain amount of the target can only release the corresponding amount of the sequence. Therefore, the SDA reaction was subsequently introduced, which used the released sequence as the template to amplify the DNA. After the hybridization of the chimera sequence with the primer, DNA polymerization was carried out when Bsm DNA polymerase and dNTP were present. Double-stranded DNA with the recognition site of the Nb.bpu10I endonuclease was produced. The Nb.bpu10I endonuclease recognized and cleavaged the recognition site, and then the strand could be extended from the cleavage site by the Bsm DNA polymerase. Because of the strong strand displacement effect of the Bsm DNA polymerase, the single strand at the downstream of the cleavage site was thus displaced from the duplex and released to the solution as the reporter probe. After multiple cycles of SDA, a large number of the reporter probes were produced and the detection signal was amplified. After the SDA reaction, a molecular beacon sequence modified with 5′FAM and 3′dabcyl was added into the system, which could be opened by the reporter probe through hybridization. Therefore, the fluorescence signal of the FAM was recovered, which was in correlation with the amount of the reporter probe and thus the content of ATP.

### 3.3. Verification of the Principle of the Aptasensor

PAGE and fluorescent spectroscopy were employed to characterize the process of biosensing and thus verify the practicability.

**Figure 3:**
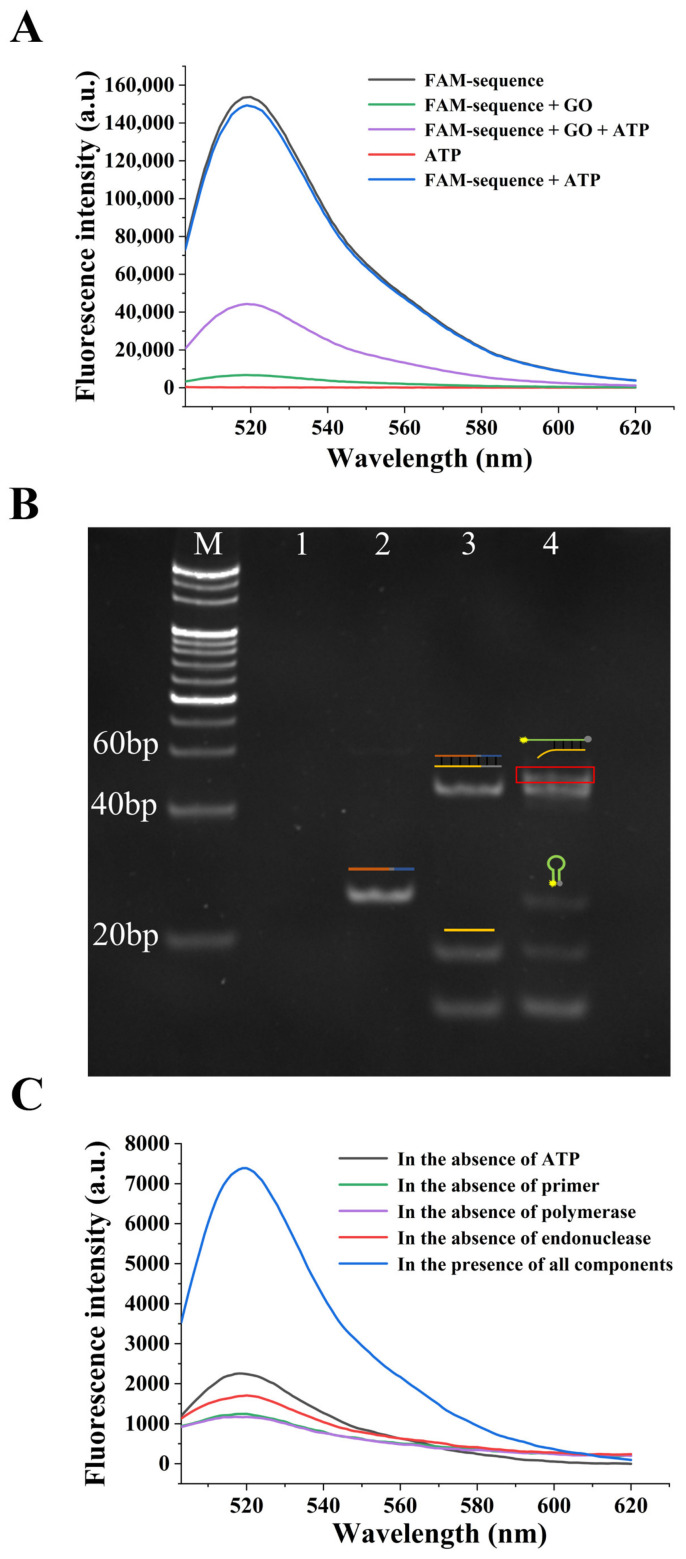
Verification of the practicability of the aptasensor. (**A**) Fluorescence emission spectra obtained in different conditions. The concentrations of the FAM-sequence and ATP were 2 μM and 1 mM, respectively. The amount of graphene oxide (GO) was 5 μL. (**B**) Polyacrylamide gel electrophoresis (PAGE) image for verification of the results under various experimental conditions. Lane M: DL20 DNA marker; lane 1: 2 μM chimera sequence and 5 μL GO; lane 2: 2 μM chimera sequence, 5 μL GO and 1 mM ATP; lane 3: 2 μM chimera sequence, 5 μL GO, 1 mM ATP, 3 μM primer, 5 U Bsm DNA polymerase, 8 U Nb.bpu10I endonuclease and 250 μM deoxyribonucleoside triphosphate (dNTP); lane 4: 2 μM chimera sequence, 5 μL GO, 1 mM ATP, 3 μM primer, 5 U Bsm DNA polymerase, 8 U Nb.bpu10I endonuclease, 250 μM dNTP and 1 μM molecular beacon. (**C**) Fluorescence spectra of various experimental and control groups. The FAM-sequence concentration was 2 μM, the amount of GO was 5 μL, the concentration of ATP was 1 mM, the concentration of primer was 3 μM, the amounts of Bsm DNA polymerase and Nb.bpu10I endonuclease were 5 U and 8 U, respectively, the concentration of dNTP was 250 μM, and the concentration of molecular beacon was 1 μM.

The fluorescence spectra under the different conditions were firstly used to characterize the process of the target-mediated displacement of the chimera sequence from the GO. As shown in Figure 3A, the 5′FAM-labeled chimera sequence exhibited a strong fluorescence intensity due to the fluorescent character of FAM; however, when the GO was added, the fluorescence intensity reduced significantly. The fluorescence was quenched effectively due to the adsorption of the sequence on the GO. When the ATP was added, the fluorescence partly recovered, indicating that the ATP could bind to the sequence and dissociate it from the GO. As the control, the ATP itself did not show any fluorescence emission, and the fluorescence intensity of the 5′FAM-labeled sequence was not affected by the ATP.

PAGE was then used to analyze the products under the different experimental conditions. As displayed in Figure 3B, when ATP was absent, the single-stranded chimera sequence was almost adsorbed by the GO, thus, the supernatant showed no band in lane 1; however, when ATP was added, the chimera sequence was dissociated from the GO, due to the displacement effect caused by the specific binding of ATP to the aptamer region, thus, a band representing the chimera sequence could be observed in lane 2. After the further introduction of the primer and two enzymes, the SDA reaction was performed, so that the bands representing the formed double-stranded product of polymerization and the displaced reporter probe could be, respectively, observed in lane 3. After the addition of the molecular beacon, the double-stranded hybridized product between the partially complementary molecular beacon and the reporter probe could be further observed in lane 4. These results prove the successful formation of the products in each step.

At the same time, the fluorescence spectra of the experimental group and different control groups were used to validate the principle. As depicted in Figure 3C, the SDA reaction could not be triggered in the absence of either the ATP, primer, Bsm DNA polymerase or Nb.bpu10I endonuclease. Therefore, the fluorescence intensities of the control groups were all significantly lower than the experimental group. All these results verify the sensing strategy principle and indicate the feasibility of the aptasensor.

### 3.4. Optimization of the Experimental Conditions

Several key factors affecting the detection have been optimized to achieve the best detection results, including the concentration of the chimera sequence and primer, the amount of Bsm DNA polymerase and Nb.bpu10I endonuclease, the time of the SDA, the concentration of molecular beacon, and the incubation time of the molecular beacon with the reporter probe. (F − F_0_)/F_0_ was employed as the index for optimization, where F and F_0_ are the fluorescence intensities of the experimental group and the control group without ATP, respectively. For all experiments, the concentration of ATP was kept constant at 1 mM. When one experiment parameter was optimized, the obtained optimal condition was then used for the subsequent experiments.

**Figure 4:**
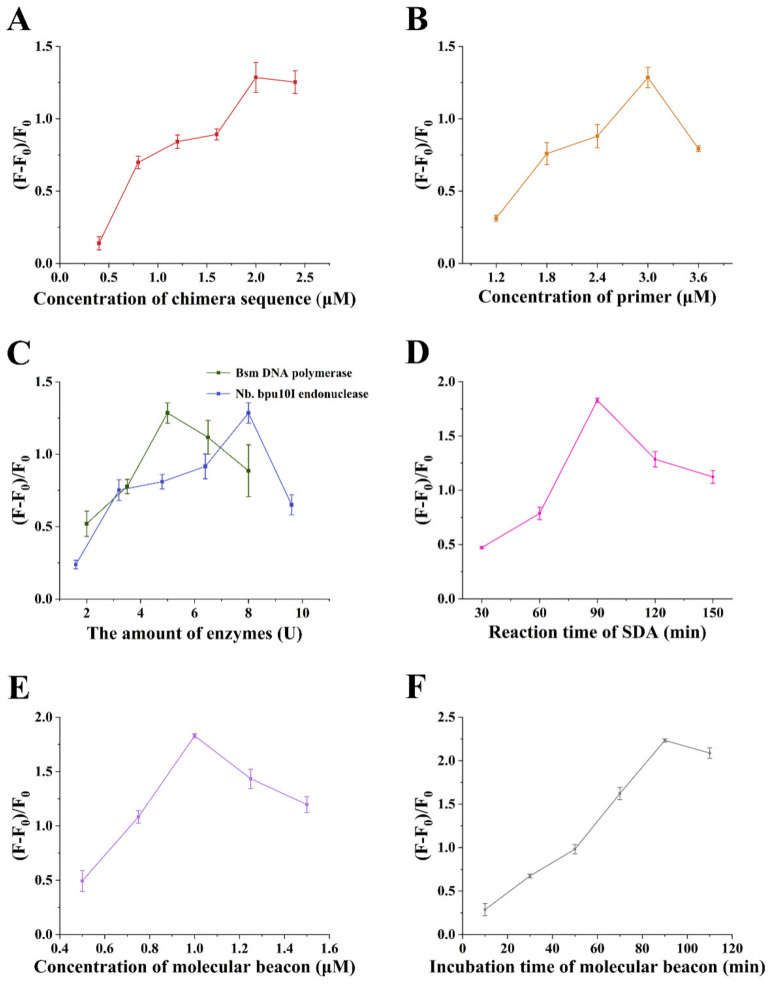
Optimization of the experimental conditions. (**A**) concentration of chimera sequence; (**B**) concentration of primer; (**C**) the amount of Bsm DNA polymerase and Nb.bpu10I endonuclease; (**D**) reaction time of strand displacement amplification (SDA); (**E**) concentration of molecular beacon; and (**F**) incubation time of molecular beacon.

The chimera sequence responds to the target and serves as the template in the SDA process, therefore, its concentration is critical to the detection. If the concentration is too low, few sequences can be released to the supernatant after incubation with ATP to start the subsequent SDA reaction. Too high a concentration will cause a high background signal. Therefore, keeping the other experimental conditions unchanged, the chimera sequence within 0.4–2.4 μM was used and the results can be seen in Figure 4A. (F − F_0_)/F_0_ continued to rise as the growing concentration of the sequence and reached the maximum at 2 μM. Therefore, a 2 μM chimera sequence was used for the subsequent experiments.

In the process of the SDA reaction, the primer hybridized with the chimera sequence and under the function of the Bsm DNA polymerase, the DNA strand was extended. The primer concentration was optimized within 1.2–3.6 μM to achieve the optimum detection performance. (F − F_0_)/F_0_ reached the maximum in the presence of a 3 μM primer (Figure 4B). Thereafter, the value decreased with a further increasing of the primer concentration, indicating that the background signal was enhanced due to the high primer concentration. Therefore, a 3 μM primer was used in the subsequent experiments.

During the SDA reaction, there was a synergistic effect between the Bsm DNA polymerase and the Nb.bpu10I restriction endonuclease. They also determined the SDA amplification efficiency, thus, they were optimized within 2–8 U and 1.6–9.6 U, respectively. As shown in Figure 4C, the (F − F_0_)/F_0_ obtained the maximum value at 5 U Bsm DNA polymerase and 8 U Nb.bpu10I endonuclease, respectively. Therefore, these amounts of the enzymes were selected for the subsequent experiments.

Meanwhile, the SDA reaction time was also one of the important factors affecting the SDA efficiency. The fluorescence intensity was measured every half an hour within 30–150 min to determine the optimal reaction time. The results can be seen in Figure 4D. As the prolongation of the reaction time, the (F − F_0_)/F_0_ increased continuously and obtained the maximum in 90 min. The best SDA time was determined to be 90 min.

The molecular beacon modified with the 5′FAM group and 3′dabcyl group served as the output signal element. The hairpin structure of the molecular beacon was opened when the reporter probe was generated, therefore the fluorescence intensity of the FAM was recovered, due to the partially complementary sequence of the reporter probe to the molecular beacon. The concentration of the molecular beacon was optimized between 0.5–1.5 μM. As shown in Figure 4E, the (F − F_0_)/F_0_ was maximized when the concentration of the molecular beacon was 1 μM.

The incubation time of the molecular beacon with the reporter probe also influenced the final signal. The fluorescence intensity was measured every 20 min from 10 min to 110 min. As shown in Figure 4F, the (F − F_0_)/F_0_ gradually rose and reached the maximum in 90 min, and then dropped slightly. Therefore, the optimal incubation time was 90 min.

### 3.5. Performance of the Fluorescent Aptasensor

**Figure 5:**
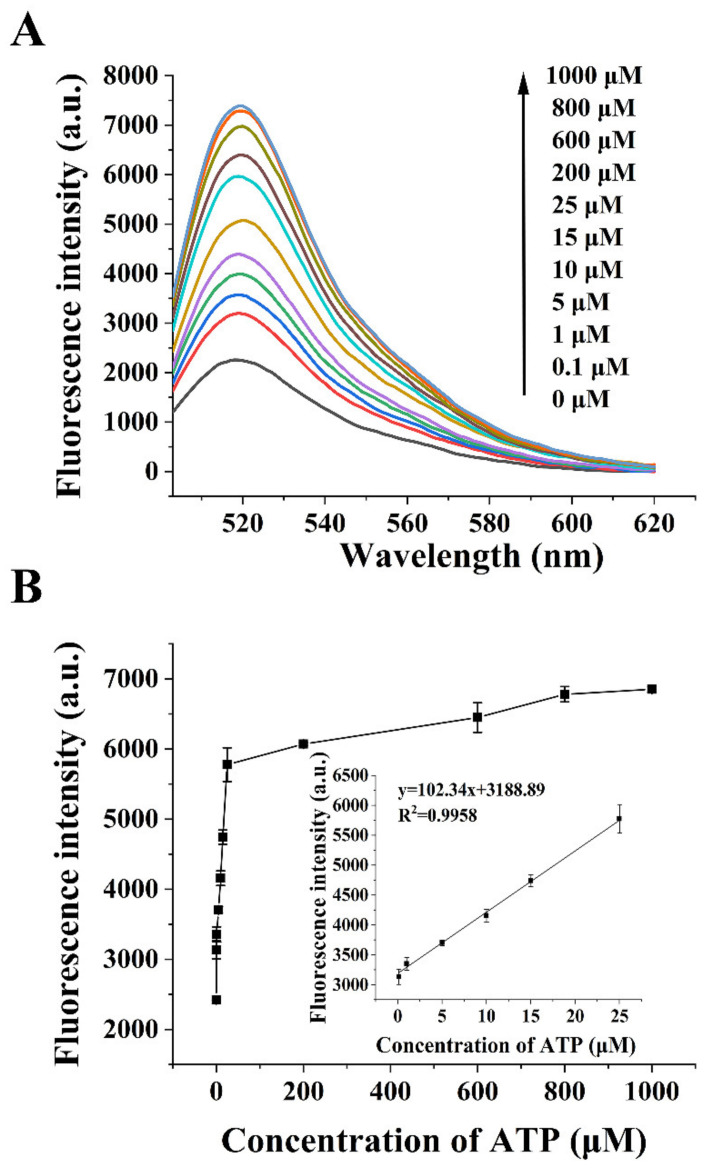
(**A**) fluorescence emission spectra in the presence of different concentrations of ATP; (**B**) the fluorescence intensity in relation to the ATP concentration. The linear relationship is shown in the inset.

The performance of the fluorescent aptasensor was investigated under the optimal detection conditions. The addition of various concentrations of ATP dissociated the chimera sequences adsorbed on the GO surface. The SDA reaction was performed following a hybridization with the primer. The generated reporter probe was used to open the molecular beacon, thereby restoring the fluorescent signal. The fluorescence emission spectra of the reaction system were recorded. The results can be seen in Figure 5A. The fluorescence intensity increased continuously with the increasing ATP concentration within 0–1000 μM. When the ATP concentration was between 0.1 μM and 25 μM, the fluorescence intensity was linearly related to the ATP concentration. The results are displayed in Figure 5B. The linear regression equation is y=102.34x+3188.89 (R^2^ = 0.9958), where x and y are the ATP concentration (μM) and the fluorescence intensity, respectively. The detection limit of this aptasensor was 33.85 nM (signal-to-noise ratio was 3). The method has a wide dynamic response range and can realize the sensitive detection of ATP.

**Figure 6:**
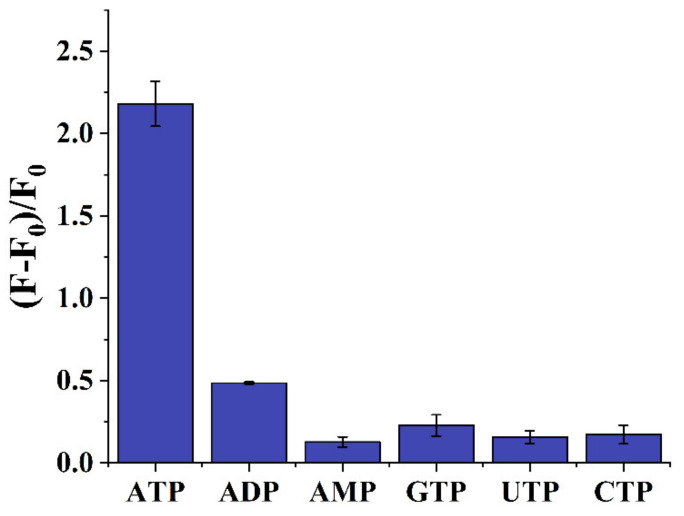
Verification of the specificity of the sensor. ATP and its analogues were fixed at 1 mM.

To test the specificity of the aptasensor, ATP analogs such as ADP, AMP, GTP, UTP and CTP were used as the control groups and detected under the same conditions. As displayed in Figure 6, in the presence of ATP, the (F − F_0_)/F_0_ value was apparently higher than the other control groups, i.e., the fluorescence aptasensor had a high specificity for the target ATP. More importantly, because of the high specificity of the newly screened aptamer, the aptasensor could easily distinguish the ATP from the ADP and AMP, thus, it had the ability to evaluate the energy charge level of the organisms. The performance of the aptasensor was compared with the commercially available ATP detection methods and kits (Table S3). Generally, it exhibited a lower detection limit.

### 3.6. Detection of ATP in Serum Samples

Different concentrations of ATP were added to human serum samples for detection in order to study the practicability of the aptasensor in actual samples. The serum samples were diluted and then detected in the same way as the standard samples. The detection recovery was within 98.745–104.676% and the RSD was within 1.978–2.961%, as shown in Table 1. The results show that the serum had a negligible impact on the detection, thus the sensor has application potential.

## 4. Conclusions

In conclusion, through magnetic beads-based SELEX, an aptamer specific for ATP was screened and then truncation optimized. This aptamer can discriminate ATP from ADP and AMP. Then, a fluorescent aptamer-based biosensor for ATP was developed employing this aptamer. The sensor converts the ATP detection to a fluorescence intensity measurement based on the combined mechanism of a target-induced DNA dissociation from GO, the SDA reaction and the switching of the molecular beacon. With a finely designed chimera sequence and efficient signal amplification mechanism, highly sensitive detection can be achieved. Particularly, the sensor has a high specificity and good performance whether in standard solutions or in real samples. As a result, the screened aptamer and the constructed biosensor have promising applications in the evaluation of the energy levels in organisms and other biological and energy-related processes.

## Figures and Tables

**Table 1:** ATP detection in human serum samples.

Samples	Added (μM)	Detected (μM)	RSD (%)	Recovery (%)
1	0.1	0.102	2.961	102.047
2	2	1.975	1.978	98.745
3	20	19.898	2.576	99.494
4	25	26.169	2.958	104.676

## Data Availability

Data is contained within the article or supplementary material.

## Supplementary Materials

The following supporting information can be downloaded at: https://www.mdpi.com/article/10.3390/s22072425/s1, Methods: Aptamer selection; Table S1: All DNA sequences used in this work; Table S2: The significance test of the affinities of the screened aptamer for ATP, ADP and AMP; Table S3: The comparison of the performance between the aptasensor in this study and the commercially available methods [20].

## References

[1] Peng L., Zhou J., Liu G., Yin L., Ren S., Man S., Ma L. (2020). CRISPR-Cas12a based aptasensor for sensitive and selective ATP detection. Sensor. Actuat. B Chem..

[2] Ratajczak K., Lukasiak A., Grel H., Dworakowska B., Jakiela S., Stobiecka M. (2019). Monitoring of dynamic ATP level changes by oligomycin-modulated ATP synthase inhibition in SW480 cancer cells using fluorescent “On-Off” switching DNA aptamer. Anal. Bioanal. Chem..

[3] Doenst T., Nguyen T.D., Abel E.D. (2013). Cardiac metabolism in heart failure: Implications beyond ATP production. Circ. Res..

[4] Ma C., Lin C., Wang Y., Chen X. (2016). DNA-based ATP sensing. TrAC-Trend. Anal. Chem..

[5] Branchini B.R., Southworth T.L., Fontaine D.M., Kohrt D., Talukder M., Michelini E., Cevenini L., Roda A., Grossel M.J. (2015). An enhanced chimeric firefly luciferase-inspired enzyme for ATP detection and bioluminescence reporter and imaging applications. Anal. Biochem..

[6] Xue X., Wang F., Zhou J., Chen F., Li Y., Zhao J. (2009). Online cleanup of accelerated solvent extractions for determination of adenosine 5′-triphosphate (ATP), adenosine 5′-diphosphate (ADP), and adenosine 5′-monophosphate (AMP) in royal jelly using high-performance liquid chromatography. J. Agric. Food Chem..

[7] Zinellu A., Sotgia S., Scanu B., Pisanu E., Sanna M., Franca Usai M., Deiana L., Carru C. (2010). Ultra-fast adenosine 5′-triphosphate, adenosine 5′-diphosphate and adenosine 5′-monophosphate detection by pressure-assisted capillary electrophoresis UV detection. Electrophoresis.

[8] Khlyntseva S.V., Bazel Y.R., Vishnikin A.B., Andruch V. (2009). Methods for the determination of adenosine triphosphate and other adenine nucleotides. J. Anal. Chem..

[9] Nimjee S.M., White R.R., Becker R.C., Sullenger B.A. (2017). Aptamers as Therapeutics. Annu. Rev. Pharmacol. Toxicol..

[10] Huang R., Xi Z., He N. (2015). Applications of aptamers for chemistry analysis, medicine and food security. Sci. China Chem..

[11] Suaebah E., Naramura T., Myodo M., Hasegawa M., Shoji S., Buendia J.J., Kawarada H. (2017). Aptamer-Based Carboxyl-Terminated Nanocrystalline Diamond Sensing Arrays for Adenosine Triphosphate Detection. Sensors.

[12] Ma L., Kartik S., Liu B., Liu J. (2019). From general base to general acid catalysis in a sodium-specific DNAzyme by a guanine-to-adenine mutation. Nucleic Acids Res..

[13] Cui W., Hu G., Lv E., Li C., Wang Z., Li Q., Qian Z., Wang J., Xu S., Wang R. (2022). A label-free and enzyme-free fluorescent aptasensor for amplified detection of kanamycin in milk sample based on target-triggered catalytic hairpin assembly. Food Control..

[14] Yazdian-Robati R., Bayat P., Oroojalian F., Zargari M., Ramezani M., Taghdisi S.M., Abnous K. (2020). Therapeutic applications of AS1411 aptamer, an update review. Int. J. Biol. Macromol..

[15] Lorenzo-Gomez R., Miranda-Castro R., de Los Toyos J.R., de-Los-Santos-Alvarez N., Lobo-Castanon M.J. (2022). Aptamers targeting a tumor-associated extracellular matrix component: The human mature collagen XIα1. Anal. Chim. Acta.

[16] Wu X., Liu H., Han D., Peng B., Zhang H., Zhang L., Li J., Liu J., Cui C., Fang S. (2019). Elucidation and Structural Modeling of CD71 as a Molecular Target for Cell-Specific Aptamer Binding. J. Am. Chem. Soc..

[17] Zheng M., Kang Y., Liu D., Li C., Zheng B., Tang H. (2020). Detection of ATP from “fluorescence” to “enhanced fluorescence” based on metal-enhanced fluorescence triggered by aptamer nanoswitch. Sensor. Actuat. B Chem..

[18] Wu X., Chen J., Wu M., Zhao J.X. (2015). Aptamers: Active targeting ligands for cancer diagnosis and therapy. Theranostics.

[19] Sassanfar M., Szostak J.W. (1993). An Rna Motif That Binds Atp. Nature.

[20] Huizenga D.E., Szostak J.W. (1995). A DNA aptamer that binds adenosine and ATP. Biochemistry.

[21] Li Y., Liu B., Huang Z., Liu J. (2020). Engineering base-excised aptamers for highly specific recognition of adenosine. Chem. Sci..

[22] Biniuri Y., Albada B., Willner I. (2018). Probing ATP/ATP-Aptamer or ATP-Aptamer Mutant Complexes by Microscale Thermophoresis and Molecular Dynamics Simulations: Discovery of an ATP-Aptamer Sequence of Superior Binding Properties. J. Phys. Chem. B.

[23] Li Y., Liu J. (2020). Aptamer-based strategies for recognizing adenine, adenosine, ATP and related compounds. Analyst.

[24] Cai S., Wang J., Li J., Zhou B., He C., Meng X., Huang J., Wang K. (2021). A self-assembled DNA nanostructure as a FRET nanoflare for intracellular ATP imaging. Chem. Commun..

[25] Ziolkowski R., Jarczewska M., Gorski L., Malinowska E. (2021). From Small Molecules Toward Whole Cells Detection: Application of Electrochemical Aptasensors in Modern Medical Diagnostics. Sensors.

[26] Shaban S.M., Kim D.H. (2021). Recent Advances in Aptamer Sensors. Sensors.

[27] Cai R., Zhang Z., Chen H., Tian Y., Zhou N. (2021). A versatile signal-on electrochemical biosensor for Staphylococcus aureus based on triple-helix molecular switch. Sensor. Actuat. B Chem..

[28] Yao J., Yue T., Huang C., Wang H. (2021). A magnified aptamer fluorescence sensor based on the metal organic frameworks adsorbed DNA with enzyme catalysis amplification for ultra-sensitive determination of ATP and its logic gate operation. Bioorg. Chem..

[29] Zheng J., Li X., Wang K., Song J., Qi H. (2020). Electrochemical Nanoaptasensor for Continuous Monitoring of ATP Fluctuation at Subcellular Level. Anal. Chem..

[30] Lu S., Hu T., Wang S., Sun J., Yang X. (2017). Ultra-Sensitive Colorimetric Assay System Based on the Hybridization Chain Reaction-Triggered Enzyme Cascade Amplification. ACS Appl. Mater. Inter..

[31] Zeng R., Luo Z., Su L., Zhang L., Tang D., Niessner R., Knopp D. (2019). Palindromic Molecular Beacon Based Z-Scheme BiOCl-Au-CdS Photoelectrochemical Biodetection. Anal. Chem..

[32] Shi K., Dou B., Yang J., Yuan R., Xiang Y. (2016). Cascaded strand displacement for non-enzymatic target recycling amplification and label-free electronic detection of microRNA from tumor cells. Anal. Chim. Acta.

[33] Xu L., Jiang Z., Mu Y., Zhang Y., Zhan Q., Cui J., Cheng W., Ding S. (2018). Colorimetric assay of rare disseminated tumor cells in real sample by aptamer-induced rolling circle amplification on cell surface. Sensor. Actuat. B Chem..

[34] Li R., Liu Q., Jin Y., Li B. (2020). Sensitive colorimetric determination of microRNA let-7a through rolling circle amplification and a peroxidase-mimicking system composed of trimeric G-triplex and hemin DNAzyme. Microchim. Acta.

[35] Hou T., Li W., Liu X., Li F. (2015). Label-Free and Enzyme-Free Homogeneous Electrochemical Biosensing Strategy Based on Hybridization Chain Reaction: A Facile, Sensitive, and Highly Specific MicroRNA Assay. Anal. Chem..

[36] Ge L., Wang W., Sun X., Hou T., Li F. (2016). Affinity-Mediated Homogeneous Electrochemical Aptasensor on a Graphene Platform for Ultrasensitive Biomolecule Detection via Exonuclease-Assisted Target-Analog Recycling Amplification. Anal. Chem..

[37] Wang Y., Yao L., Ning G., Wu Y., Wu S., Mao S., Liu G.Q. (2019). An electrochemical strategy for tetracycline detection coupled triple helix aptamer probe with catalyzed hairpin assembly signal amplification. Biosens. Bioelectron..

[38] Cai R., Yin F., Chen H., Tian Y., Zhou N. (2020). A fluorescent aptasensor for *Staphylococcus aureus* based on strand displacement amplification and self-assembled DNA hexagonal structure. Microchim. Acta.

[39] Yue H., Chen J., Chen X., Wang X., Zhang Y., Zhou N. (2021). Systematic screening and optimization of single-stranded DNA aptamer specific for N-acetylneuraminic acid: A comparative study. Sensor. Actuat. B Chem..

