# Supplementary material for: Fluorescent Aptasensor for Highly Specific Detection of ATP Using a Newly Screened Aptamer
Source: Sensors (Basel). 2022 Mar 22;22(7):2425. doi: 10.3390/s22072425 (PMC9003339; doi:10.3390/s22072425)
Supplement: Supplementary file 1 [file sensors-22-02425-s001.zip › sensors-1634391-supplementary.pdf]

Supplementary Information

Fluorescent aptasensor for highly specific detection of ATP using a newly screened aptamer

Xin Chen[1,#], Yangkun Feng[2,#], Haohan Chen[1], Yuting Zhang[1], Xiaoli Wang[1] and Nandi Zhou[1,*]

[1] The Key Laboratory of Carbohydrate Chemistry and Biotechnology, Ministry of Education, School of Biotechnology, Jiangnan University, Wuxi 214122, China

[2] Medical College of Nantong University, Nantong 226007, China

[#] These authors contribute equally.

[*] Correspondence: zhounandi@jiangnan.edu.cn (N.Z.); Tel.: +86-510-85197831

Aptamer selection

Screening of ATP-specific aptamers was performed using DNA library-immobilized magnetic beads-based SELEX. PCR amplification of the initial library was performed using primer1 and primer2. The PCR amplification system includes 9.5 μL ddH_2O, 12.5 μL 2×PrimeSTARMax Premix, 1 μL primer1 (10 μM), 1 μL primer2 (10 μM), with 1 μL initial ssDNA library (1 μM). The PCR amplification was carried out at 95°C, 5 min; 95°C, 30 s; 55°C, 30 s; 72°C, 15 s; 72°C, 5 min; 15 cycles. In order to retain the activity of the streptavidin-modified magnetic beads, they were washed 5 times with 1 mL of 10 mM PBS buffer (137 mM NaCl, 2.7 mM KCl, pH 7.4) for 5 min before each use. The washed magnetic beads can be stored at 4°C in the refrigerator. During the first round of the screening, 100 μL of PCR product was diluted with 100 μL of binding buffer (50 mM Tris-HCl, 5 mM KCl, 100 mM NaCl, 1 mM $MgCl_2$, pH 7.4), followed by addition of 50 μL activated magnetic beads. After two hours of gentle shaking at room temperature, the mixture was placed on a magnetic separator. The supernatant was discarded, and the binding buffer was used to wash the beads 5 times to eliminate the double-stranded DNA uncoupled on the beads. Subsequently, 3 μL of 100 mM ATP was added and continued to incubate for 2 h at 25°C, followed by placing on a magnetic separator for 3 min. And the supernatant was collected as the secondary library for the next screening round. As the number of screening rounds increased, the concentration of ATP was gradually reduced to increase the screening pressure in order to get high-affinity aptamers. At the same time, in order to improve the specificity of aptamers, counter screening was

introduced since the seventh round. During counter screening, ADP and AMP with the same concentration of ATP were added and incubated with double-stranded DNA-coupled magnetic beads for 2 h. Following magnetic separation, the supernatant was eliminated and then ATP was added for further incubation. After three rounds of continuous counter screening and one round of positive screening, the selection was finished.

Table S1. All DNA sequences used in this work.

Name	Sequences (5'-3')
Initial ssDNA library	TAGGGAATTCGTCGACGGATCC-N35-CTGCAGGTC GACGCATGCGCCG
Primer1	TAGGGAATTCGTCGACGGAT
Primer2	biotin-CGGCGCATGCGTCGACCTG
ATP aptamer	TGGCGTCTGCATGCAGGTCGACGCATGCGCCG
Classic ATP aptamer	ACCTGGGGGAGTATTGCGGAGGAAGGT [1]
Functional chimera sequence	*TGGCGTCTGCATGCAGGTCGACGCATGCGCCG***CCT GAGC**TTAGAT
FAM-labeled functional chimera sequence	FAM-TGGCGTCTGCATGCAGGTCGACGCATGCGC CGCCTGAGCTTAGAT
Primer3	ATCTAAGC
Molecular beacon	FAM-TCAGGCGGCGTTTTTTTTGGTCGACGCATGC GCCGCCTGA-dabcyl

The italicized letters in the functional chimera sequence represent the aptamer region and the bold letters represent the complementary sequence of the Nb.bpu10I endonuclease cleavage site. The underlined letters represent the complementary sequences of the primer.

Table S2. The significance test of the affinities of the screened aptamer for ATP, ADP and AMP.

Descriptives

	N	Mean	Std. Deviation	Std. Error	95% Confidence Interval for Mean		Minimum	Maximum
					Lower Bound	Upper Bound		
ATP	3	161.6667	10.50397	6.06447	135.5734	187.7600	151.00	172.00
ADP	3	67.6667	6.42910	3.71184	51.6959	83.6374	63.00	75.00
AMP	3	34.6667	6.65833	3.84419	18.1265	51.2069	27.00	39.00
Total	9	88.0000	57.49565	19.16522	43.8049	132.1951	27.00	172.00

Descriptives is a statistical method to conduct the basic information statistics of nine data in three groups of ATP, ADP and AMP, including the number of examples, mean, standard deviation and other information for each group.

ANOVA

	Sum of Squares	df	Mean Square	F	Sig.
Between Groups	26054.000	2	13027.000	199.393	.000
Within Groups	392.000	6	65.333		
Total	26446.000	8			

ANOVA is a statistical method to analyze whether the data between each group is significantly different. Under the assumption of homogeneity of variance, within the 95% confidence interval, the difference between the affinities of the screened aptamer for ATP, ADP and AMP is statistically significant ($F = 199.393$, $P < 0.001$).

Table S3. The comparison of the performance between the aptasensor in this study and the commercially available methods.

Name	Method	Sensitivity	Selectivity	Manufacturer
The aptasensor in this study	Fluorescent	33.85 nM	Good	
PhosphoWorks™ Fluorimetric ATP Assay Kit	Bioluminescence	0.4 μM	Not reported	AAT Bioquest
ATP Assay Kit	Colorimetric/Bioluminescence	1 μM	Not reported	Abcam
ATP Bioluminescence Kit	Bioluminescence	1 nM-10 μM	Not reported	Sigama-Aldrich
ATP Bioluminescence Assay Kit HS II	Bioluminescence	10^{-12} M	Not reported	Sigama-Aldrich
ATP Assay Kit	Bioluminescence	10 μM	Not reported	Beyotime
ATP Content Assay Kit	Micromethod	2.6 μM	Not reported	Solarbio
ATP Content Assay Kit	Colorimetric	2.3 μM	Not reported	Solarbio
ATP Content Assay Kit	Colorimetric	3 μM	Not reported	Boxbio
ATP Content Assay Kit	Colorimetric	2.3 μM	Not reported	Sangon Biotech
ATP Content Assay Kit	Micromethod	2.6 μM	Not reported	Sangon Biotech

Reference

1. Huizenga, D.E.; Szostak, J.W. A DNA aptamer that binds adenosine and ATP. *Biochemistry* **1995,** *34*, 656-665, 10.1021/bi00002a033.
